# Effects of indoor cycling (spinning) on physiological, cardiac and perceived exertion responses in adults: a systematic review

**DOI:** 10.3389/fspor.2026.1715110

**Published:** 2026-07-22

**Authors:** Boryi A. Becerra-Patiño, Aura Daniela Montenegro-Bonilla, Carlos F. Martínez-Benítez, José Pino-Ortega, Sam Hernández-Jaña, Antonio Castillo-Paredes, Jorge Olivares-Arancibia, Rodrigo Yáñez-Sepúlveda, José Francisco López-Gil

**Affiliations:** 1 Faculty of Physical Education, National Pedagogical University, Bogota, Colombia; 2 Management and Pedagogy of Physical Activity and Sport (GPAFD), Faculty of Physical Education, National Pedagogical University, Bogota, Colombia; 3 Faculty of Sport Science, University of Murcia, Murcia, Spain; 4 IRyS Group, Physical Education School, Pontificia Universidad Católica de Valparaíso, Valparaíso, Chile; 5 AFySE Group, Research in Physical Activity and School Health, School of Physical Education, Faculty of Education, Universidad de Las Américas, Santiago, Chile; 6 Faculty Education and Social Sciences, Universidad Andres Bello, Viña del Mar, Chile; 7 School of Medicine, Universidad Espíritu Santo, Samborondón, Ecuador; 8 Vicerrectoría de Investigación y Postgrado, Universidad de Los Lagos, Osorno, Chile

**Keywords:** adults, heart rate, maximum oxygen consumption, physical activity, spinning

## Abstract

**Background/objectives:**

Indoor cycling (IC), also known as spinning, is a physical activity that takes place in the gym, where participants with different levels of physical condition, age, and objectives pedal on a modified stationary bike to maintain the rhythm according to the music and the instructor's cues. However, it is necessary to investigate the effects of this practice on people with low levels of physical fitness. The objective of this review was to analyze the scientific literature on the effects of indoor cycling in relation to gender and in terms of physiological indicators, cardiac parameters, and perceived exertion in adults.

**Methods:**

This systematic review follows the Preferred Reporting Items for Systematic Reviews and Meta-Analyses (PRISMA) guidelines. The following databases were consulted: PubMed (Medline), Web of Science (WoS), and Scopus. The PICO strategy was used to search for documents. To assess the risk of bias, each of the included articles was evaluated using version developed by Law et al.

**Results:**

A 20-minute IC session with an intensity of 80%–90% maximum heart rate (HRmax) confers positive effects on physiological processes. Gender, age, experience, and the use of heart rate monitors do not significantly affect HR or rating of perceived exertion (RPE), while environmental conditions such as sweat rate, in-creased temperature, and humidity significantly influence exercise intensity.

**Conclusions:**

IC was consistently associated with a high-intensity physiological demand and, in several studies, with improvements in cardiorespiratory fitness and cardiometabolic risk markers. Within this more limited evidence base, IC has also been examined in specific contexts such as cardiac rehabilitation and weight management in women with overweight, suggesting favorable metabolic adaptations, although the heterogeneity of populations and outcomes across studies warrants caution when generalizing these findings.

## 1 Introduction

Indoor cycling (IC), also known as spinning, is a physical activity that takes place in the gym, where participants with different levels of physical condition, age, and objectives pedal on a modified stationary bike to maintain the rhythm according to the rhythm of the music and the indications of the spinning trainer (1). In this type of activity, music takes center stage because it produces motivation in the participants and variability in the intensities of the exercise achieved (2). It is characterized by cardiovascular activity that involves the muscles of the lower limbs of the body (3).

The trainer plays a fundamental role in spinning because it allows the intensity associated with the music to be controlled, which means that participants must make different modifications that produce the intensity of the exercise performed (4). The monitoring of different intensity indicators, such as heart rate (HR), maximum oxygen consumption (VO_2max_), and rating of perceived exertion (RPE), allows the trainer to assess and control performance within safe ranges for each participant, thus avoiding overexertion and overtraining (5, 6) and making this practice a safe space that has health benefits (7). For this purpose, various methods have been used to quantify the intensity, with the HR being the most studied variable, mainly through heart rate monitoring, which is based on the pre-diction of the maximum HR (HRmax) (8).

The intensity produced by spinning is associated with different changes in position, sitting or standing, and cadence (9). According to Barbado & Barranco (10), the trainer has several tools to control the intensity of the exercise: 1) the cadence with which you pedal and which is directly related to the rhythm of the music; 2) the braking resistance applied by each of the practitioners; 3) the position on the bike; and 4) the relationship between the periods of work and recovery.

In this sense, trainers can adapt the intensity levels of exercise to the level of the physical fitness of practitioners. The trainer is the person trained to monitor the intensity, supervise the effect it is producing on each practitioner, safeguard the health of the person, and program the stimuli generated by the workload (11).

One of the main characteristics of cycling lies in the high-moderate intensity variability that generates adaptations in the cardiovascular system and skeletal muscles (11, 12). It has constituted a method of physical conditioning because of the multiple benefits it produces; among them, it has been reported that three months of spinning as specific training or alternating with strength work produces improvements in the aerobic capacity of practitioners and, at the same time, interventions of 6 months of spinning practice and an ad-equate diet allow a decrease in diastolic blood pressure (7). Therefore, several benefits are produced by the regular practice of spinning. The product of the similarity of the vertical movement that occurs when walking is that spinning is also used with rehabilitation and physical conditioning guidelines (13).

Other studies have analyzed the physiological effects that occur with the practice of spinning, reported the adaptive processes produced by spinning in the context of muscle fatigue among practitioners (14), adaptations of the left ventricle after 12 weeks of spinning practice in untrained women (15), effects produced by spinning in women with overweight/obesity and high levels of sedentary lifestyle (16), and comparisons of the cardiorespiratory response and cardiometabolic response of the practice of spinning with other sports, such as cross-country skiing and alpine skiing (17). Anthropometric factors, including the effects of 16 weeks of Spinning on the body composition of adolescent students, have also been studied (18).

Compared with the above, the need to continue developing studies that analyze the effects of the practice of spinning in different population groups is confirmed because the acquired knowledge is useful to favor a greater understanding of the transfer of knowledge that the trainer performs to favor the practices he develops (19) and, with it, seeks to understand the knowledge and findings to be used by practitioners. Therefore, the objective of this review was to analyze the scientific literature on the effects of Indoor cycling (spinning) in relation to gender and in terms of physiological indicators, cardiac parameters, and perceived exertion in adults, with the aim of providing relevant information to researchers, institutions, and professionals in the fields of physical activity and health.

## 2 Material and methods

### 2.1 Design

This systematic review follows the Preferred Reporting Items for Systematic Reviews and Meta-Analyses (PRISMA) guidelines (20) and the instructions for conducting systematic reviews in sports science (21). The search approach, along with the selection criteria and additional details, was previously noted in the prospective registry for INPLASY systematic reviews (INPLASY20248003).

### 2.2 Information sources

The search strategies consider the following characteristics:

Date: April 30, 2024.

The following databases were consulted: PubMed (Medline), Web of Science (WoS), and Scopus.

### 2.3 Search strategy

To design the search strategy, the P (population), I (intervention), C (comparison), and O (outcomes) approaches were applied, as suggested by the guidelines used for conducting this systematic review. The Boolean commands “AND” and “OR” were used to group the mentioned terms. A similar approach was followed for each database. Before finalizing the search string for each database, searches were tested via the following list of words: “Indoor Cycling”, “Spinning”, “physiological responses”, “Physiology”, “Anthropometric”, “Measurements”, “Effects”, “Exercise”, “Body Composition”, “Heart Rate”, “Wearables”, and “Physical Fitness”.

The review of the different searches was independently conducted by two authors to define which terms provided the highest-quality documents on the topic. As a result, the selected terms were “Cycling Indoor”, “Spinning”, “Anthropometric”, “Physiology*”, “Physiological responses”, “Effect*”, “Exercise”, and “Physical Fitness”. Based on these terms, the following search equation was constructed: (“Indoor Cycling” OR Spinning) AND (Anthropometric OR Physiology*) AND (“Physiological responses” OR Effect* OR Exercise OR “Physical Fitness” OR Body Composition). This search string was adapted for use in the PubMed, WoS, and Scopus databases. Controlled vocabulary searching was combined with keyword searching to improve retrieval.

Searches were conducted to identify studies without other restrictions regarding publication date, language, or study design. Additionally, citation searches were performed for the key studies. When full-text articles could not be obtained through institutional subscriptions or open access, an attempt was made to contact the corresponding authors directly.

### 2.4 Inclusion and exclusion criteria

The inclusion criteria were as follows: (i) published studies with no language restrictions; (ii) original studies; (iii) quantitative studies; (iv) studies that explicitly examined and analyzed the effects of spinning in adult population (18 years of age or older); (v) studies with no restrictions on methodological design; (vi) studies that use spinning and draw comparisons with other types of populations. Both descriptive and experimental studies were included. The exclusion criteria were as follows: (i) abstracts, conference proceedings, books, book chapters, and letters to the editor; (ii) articles not peer-reviewed; (iii) studies without full access to the original text; (iv) gray literature; (v) systematic reviews, meta-analyses, bibliometric analyses, narrative reviews, or literature reviews.

The search was conducted by two authors, who identified the most relevant information from each study. Subsequently, the data (title, authors, journal, date, and database) were imported into an Excel spreadsheet, where duplicates were identified and removed. If any relevant studies were found outside the search strategy, they were added as “external sources.” The selection and inclusion of studies in this review were based on the inclusion and exclusion criteria derived from the PICO strategy (Table 1).

**Table 1:** Inclusion and exclusion criteria based on the PICO strategy.

Topic	Inclusion	Exclusion	Search coherence
Population	Spinning participants aged 18 and older	Spinning participants under the age of 18	(Indoor Cycling OR Spinning)
Intervention	Evaluation of at least one physiological, physical or anthropometric condition variable	Evaluation restricted exclusively to psychological variables, or to biomechanical variables not linked to physiological, physical, or anthropometric outcomes.	
Outcomes	Results that relate physiological indicators (cardiorespiratory fitness (CF), cardiac output, VO_2max_), profiles of physical abilities (e.g., potency, explosive strength, cardiovascular fitness), subjective (subjective perception of effort), and biomechanical indicators directly related to exercise intensity, performance, or safety (e.g., pedaling cadence, body position, power output, saddle adjustment)	Results that do not relate physiological indicators, profiles of physical abilities, subjective.	(Anthropometric OR Physiolog*) AND (“Physiological responses” OR Effect* OR Exercise OR “Physical Fitness” OR Body Composition)
Other critics	Original research that has been reviewed by academic peers	Systematic reviews, meta-analysis, bibliometric analysis, narrative reviews, literature. Patents, abstracts, meetings, books, reviews, letters and editorials; iv) validation of instruments; v articles written without peers, studies without full access to the original text	–

### 2.5 Data extraction

After the identification of the studies, the documents were downloaded in Excel from the following data for each study: i) title; ii) authors; iii) journal; iv) year; and v) database. Once the documents of each database were downloaded, a single database was unified. Similarly, it is worth mentioning that it did not appear in the search strategy; rather, it was added through external sources. The process was carried out independently by two of the authors (“AM-B” and “BAB-P”). Any disagreement (5% of the total documents) on the final state of inclusion-exclusion was resolved through academic discussion, both in the selection phase and in the exclusion phase. In the discussion process, the two independent authors analyzed the articles at the same time following the criteria set out in the order of Table 1. This process is systematized in Excel. Thus, to continue the PRISMA guidelines, data extraction oversaw “AM-B” and “BAB-P”.

### 2.6 Methodological quality

To assess the risk of bias, each of the included articles was evaluated via an adapted version (22) of the original version developed by Law et al. (23). This scale is composed of 16 items, as detailed in Table 2.

**Table 2:** Methodological quality of studies.

Reference	1	2	3	4	5	6	7	8	9	10	11	12	13	14	15	16	Score
Caria et al. (11)	1	1	1	1	0	1	1	1	1	1	0	1	0	1	1	0	12/16
Battista et al. (12)	1	1	1	1	1	1	1	1	1	1	1	1	0	1	1	1	15/16
de Melo Dos Santos et al. (14)	1	1	1	1	1	1	1	1	1	1	1	0	0	1	0	0	12/16
Bianco et al. (16)	1	1	1	1	0	1	0	1	1	1	1	1	0	1	1	0	12/16
Lai et al. (24)	1	1	1	1	1	0	0	1	1	1	1	1	1	1	1	1	14/16
Cortis et al. (25)	1	1	1	1	0	1	1	1	1	1	1	1	0	1	1	1	14/16
Ratajczak et al. (26)	1	1	1	1	1	0	1	1	1	1	1	0	1	1	1	0	13/16
Canário-Lemos et al. (27)	1	1	1	1	0	1	1	1	1	1	1	1	0	1	1	1	14/16
Barbado et al. (28)	1	1	1	1	0	1	1	1	1	1	1	1	0	1	1	0	13/16
Luszczyk et al. (29)	1	1	1	1	0	1	1	1	1	1	1	0	1	1	0	1	13/16
Rendos et al. (30)	1	1	1	1	0	1	0	0	1	1	1	1	1	0	1	1	12/16
Soriano-Maldonado et al. (31)	1	1	1	1	0	1	0	1	1	1	1	1	0	1	1	1	13/16
Ramos-Jiménez et al. (32)	1	1	1	1	0	1	0	1	1	1	1	1	0	1	1	1	13/16
Muyor (33)	1	1	1	1	0	1	1	1	1	1	1	1	0	1	1	1	14/16
Muyor & López-Miñarro (34)	1	1	1	1	0	1	1	1	1	1	1	1	1	1	0	1	14/16
López-Miñarro & Muyor (35)	1	1	1	1	0	1	1	1	1	1	1	1	0	1	1	1	14/16
Kang et al. (36)	1	1	1	1	0	1	1	1	1	1	1	0	0	1	0	0	11/16

Item 1: Was the study purpose stated clearly?; Item 2: Was relevant background literature reviewed?; Item 3: Was the design appropriate for the research question?; Item 4: Was the sample described in detail?; Item 5: Was sample size justified?; Item 6: Was informed consent obtained? (if not described, assume No); Item 7: Were the outcome measures reliable? (if not described, assume No); Item 8: Were the outcome measures valid? (if not described, assume No); Item 9: Was method described in detail?; Item 10: Were results reported in terms of statistical significance?; Item 11: Were the analysis methods appropriate?; Item 12: Was importance for the practice reported?; Item 13: Were any drop-outs reported?; Item 14: Were conclusions appropriate given the study methods?; Item 15: Are there any implications for practice given the results of the study?; Item 16: Were the limitations of the study acknowledged and described by the authors?.

### 2.7 Identification and selection of studies

**Figure 1:**
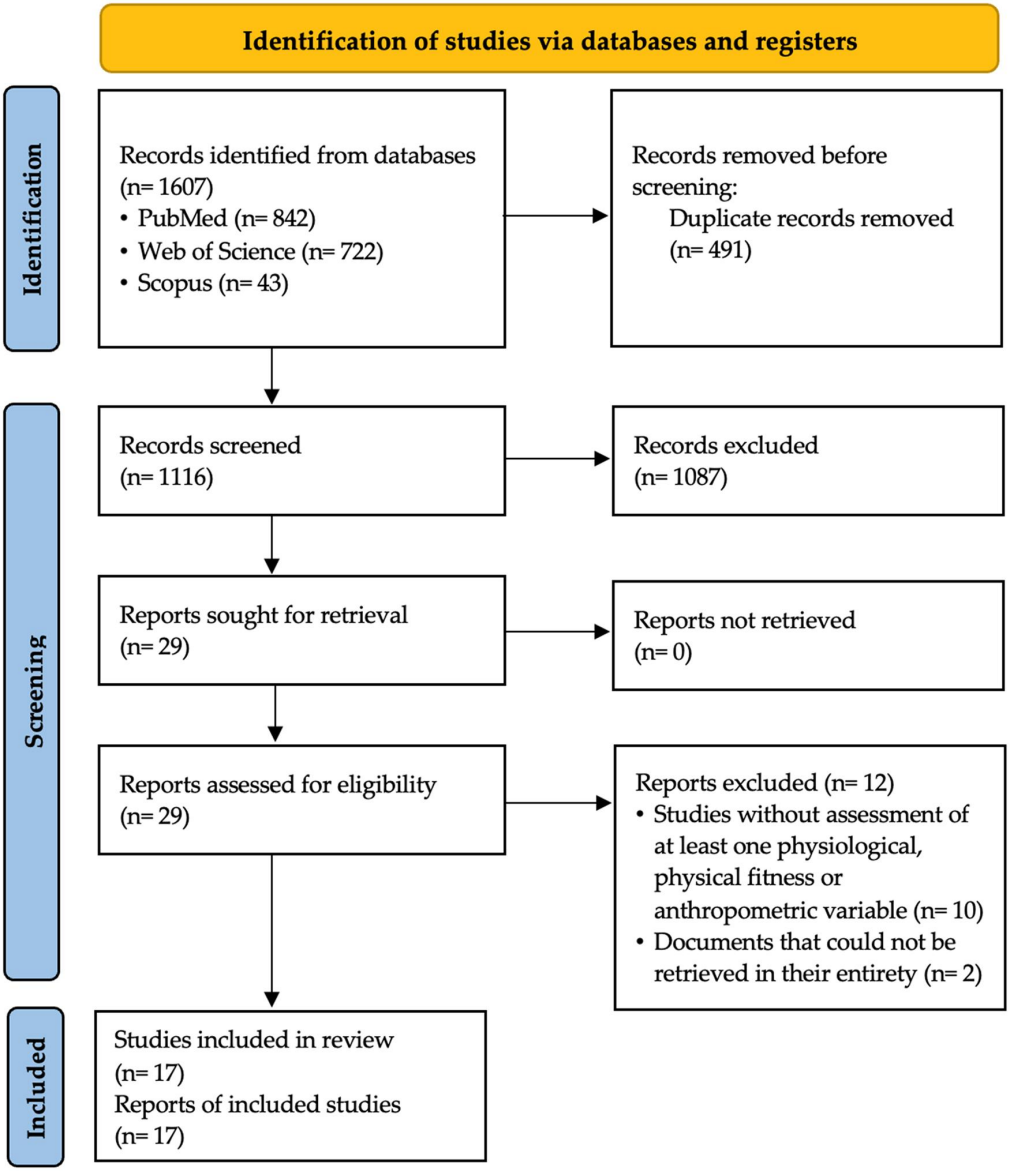
Flow diagram for selection of studies according to PRISMA guidelines.

In total, 1,607 documents were identified. After creating a single database with all the documents, we first proceeded to identify duplicate documents (*n* = 491). The remaining records were screened, and those not related to the theme were excluded (*n* = 1,087). Of the 29 documents initially selected to confirm their inclusion, all were retrieved and assessed in full for systematic reading. Thus, of the 29 eligible documents whose eligibility was analyzed in their entirety, 12 were excluded. After this process, 17 articles met all the inclusion criteria (Figure 1).

### 2.8 Key characteristics of the population samples evaluated

Table 3 outlines the main characteristics of the samples evaluated in each of the 17 included studies. It details characteristics related to the number of participants, their sex and age, and anthropometric (weight, height, BMI) and physiological variables (VO_2max_ and HRmax)_._

**Table 3:** Classification of the general variables of the selected studies.

Author (year)	Number of participants	Sex	Age (yrs)	Weight (kg)	Height (cm)	BMI (kg/m^2^)	VO_2max_ (ml·kg−^1^·min−^1^)	HRmax (bpm)
Caria et al. (2007) (11)	6 6	♂ ♀	30.0 ± 4.8 43.0 ± 6.3	74.0 ± 8.3 56.0 ± 5	177.0 ± 6.5 163.0 ± 2.6	24.0 ± 2.5	48.4 ± 4.1 42.0 ± 6.5	176.0 ± 10 175.0 ± 12
Battista et al. (2008) (12)	20	♀		64.7 ± 6.8	168.0 ± 6	24.4 ± 5.8		
de Melo Dos Santos et al. (2017) (14)	10 (Tra) 12 (Sed)	♂	23.8 ± 1.0 25.9 ± 2.3	55.5 ± 2.9 62.3 ± 2.2	167.0 ± 3.2 168.0 ± 1.8			
Bianco et al. (2010) (16)	14	♀	22.6 ± 2.1	70.8 ± 8.8 (Bef) 68.6 ± 9.2 (af 36SP-s)		25–29.9		
Lai et al. (2022) (24)	17 13	♂ ♀	24.29 ± 1.72 27.38 ± 4.0	72.0 ± 10.0 56.6 ± 6.5	176.0 ± 6.04 159.6 ± 4.2	23.18 ± 2.72 22.30 ± 3.30		
Cortis et al. (2021) (25)	10 8	♂ ♀	51.34 ± 8.02	78.4 ± 11.4 66.6 ± 3.6	180.1 ± 6.7 174.4 ± 8.5		47.6 ± 3.7 41.1 ± 4.1	193.0 ± 8.0 190.0 ± 7.4
Ratajczak et al. (2020) (26)	8 (NW) 18 (OW)	♀	47.0 ± 5.04 51.0 ± 6.66	66.0 ± 5.4 95.2 ± 19.6	165.0 ± 0.07 162.0 ± 0.06	24.3 ± 1.31 36.2 ± 5.64		
Canário-Lemos et al. (2020) (27)	12	♂	26.8 ± 5.10	77.7 ± 9.5	177.7 ± 5.6		39.75 ± 6.1	
Barbado et al. (2018) (28)	184 116	♂ ♀	41.06 ± 8.05	74.0 ± 14.4	179.6 ± 9.1	25.26 ± 3.64		
Luszczyk et al. (2018) (29)	6	♂	23.5 ± 0.71				46.8 ± 2.05	178 ± 4.1
Rendos et al. (2015) (30)	11	♂ - ♀	24.4 ± 6.4	65.7 ± 1.2	168.0 ± 0.90			
Soriano-Maldonado et al. (2014) (31)	8 8	♂ ♀	42.2 ± 8.6	75.3 ± 7.1 59.8 ± 6.5	176.0 ± 5.0 165.0 ± 5.2	24.3 ± 3.5 22.0 ± 2.1		175 ± 9.0 184 ± 6.0
Ramos-Jiménez et al. (2014) (32)	12 9	♂ ♀	30.0 ± 6.2 24.1 ± 4.5	77.4 ± 16.7 62.2 ± 2.4	175.0 ± 0.0 165.0 ± 0.0	25.2 ± 5.1 22.9 ± 1.7		
Muyor (2013) (33)	53	♂ - ♀	28.79 ± 6.0	69.5 ± 13.6	171.0 ± 0.0			
Muyor & López-Miñarro (2012) (34)	80	♂ - ♀	32.3 ± 9.5	75.6 ± 19.2	173 ± 0.0	25.2 ± 0.0		
López-Miñarro & Muyor (2010) (35)	59	♂ - ♀	32.1 ± 10.2	77.5 ± 18.5	170.0 ± 11			
Kang et al. (2005) (36)	7 8	♂ ♀	23.0 ± 1 24.0 ± 7	83.4 ± 19.1 64.2 ± 8.9	177.0 ± 8 165.0 ± 7		37.8 ± 8.2 32.2 ± 8.1	187.0 ± 9 184.0 ± 5

Yrs, years; kg, kilograms; cm, centimeters; Con, control group; Exp, experimental group; Exer, exercise; con, control; NW, normal weight; OW, obese weight; Sed, sedentary; Exe, exercise group; Tra, trained; Bef, before; af 36SP-s, after 36 spinning sessions; ♂, male; ♀, female.

### 2.9 Methodological characteristics of the included studies

Table 4 below summarizes the scientific evidence from the included studies based on an analysis of the main methodological characteristics of the research. It details the objective, the instruments used to quantify the effects of spinning, the main conclusions of the studies, and the practical applications.

**Table 4:** Classification of the methodological procedures of the studies.

Reference (year)	Objective	Instruments	Conclusions	Practical applications
Caria et al. (2007) (11)	Evaluate a series of metabolic and cardiovascular variables during a standard 50-minute class conducted by Spinning instructors.	Technique (MBIA, InBody 3.0 Biospace; cycle ergometer (Cardio-line STS400; monitored using an ergospirometry system (Metamax X1, Cortex Biophysik).	This type of physical activity has a high impact on cardiovascular function and suggests that it is not suitable for unfit or sedentary people, especially middle-aged or elderly people, who are willing to start a recreational physical activity program.	Spinning affects cardiovascular function, it is suggested that it is not suitable for sedentary people, especially older people.
Battista et al. (2008) (12)	Make observations of the intensity of Spinning, to document the intensity of the exercise during Spinning.	Open circuit spirometry (Applied Electrochemistry, Inc., Pittsburgh, PA; electrically braked cycle ergometer (Lode Excalibur, Groningen, Netherlands).	The intensity of Spinning in healthy and physically active women is moderate; there are frequent observations of transient o2 values that exceed o2max and a substantial part of exercise episodes at intensities higher than VT. As such, the data suggest that Spinning should be considered a high-intensity exercise mode of exercise training, which has implications for both effectiveness and risk.	From the point of view of the use of Spinning classes to contribute to physical conditioning. It is reasonably well established that higher-intensity training is necessary to cause adaptations to the cardiorespiratory system. Therefore, in addition to the low-impact nature of this mode of exercise, it may be that this is an effective method of nonspecific conditioning that would be very effective based on time results.
de Melo Dos Santos et al. (2017) (14)	Evaluate the differences in surface electromyography variables (sEMG), HR, and subjective effort in sedentary participants while performing a Spinning session and compare their results with trained subjects, to answer the question: Are trained cyclists less susceptible to muscle fatigue than sedentary cyclists?	Bike (Kikos; Model Pro F12); (Polar FT7, USA); (model EMG820C, Emgsystem inc; São Jose dos Campos, Brazil).	Sedentary participants are more likely to be tired, and Spinning can be incorporated into the protocols for this population, but their fitness levels must be considered because each performance depends on the physical condition of the individual.	Spinning can be incorporated into the protocols for the sedentary population, but their fitness levels must be considered because each performance depends on the physical condition of the individual.
Bianco et al. (2010) (16)	Evaluate body composition and physiological effects in overweight sedentary young women after training in Spinning.	InBody 320, Biospace (Beverly Hills, Los Angeles, CA, USA); blood pressure machine (Mx3 plus, Omron, Germany; HR monitors (S810i, Polar, Oulu, Finland); ergo spirometer system (K4 b2, Cosmed srl, Rome, Italy) and U5x upright cycle stationary bike (Matrix, Cottage Grove, OR, USA).	Decreased body weight, without any restriction on food consumption. Improved cardiorespiratory fitness suggests that Spinning can be efficient in losing weight and preventing the increased risk of cardiovascular disease in young overweight women. Spinning can be done by sedentary young overweight women.	Spinning programs for young overweight women that seek to improve cardiorespiratory fitness and reduce fat mass, without the need for strict dietary restriction, which makes it accessible and sustainable in the long term.
Lai et al. (2022) (24)	Find an indicator of the body's physical load during incremental exercise. Our goal was to investigate the relationship between SVV and HRV during incremental cycle experiments.	AESCULONTM ICG equipment (Osypka Medical, Berlin, Germany).	These findings can be used as a guide for exercise medical care. Pausing or reducing the exercise load before entering the ACP (aerobic capacity plateau) could reduce the risk of myocardial injury.	These conclusions suggest that SVV and HRV analysis can be a valuable tool for monitoring physical load during exercise and avoiding overtraining, which has important applications for health care and the prevention of myocardial injuries.
Cortis et al. (2021) (25)	Determine the effects of interval intensity sequencing on energy expenditure (EE), physiological markers, and perceptual responses during Spinning.	Electronically braked cycle ergometer (Lode Excalibur, Groningen, Netherlands).	Interval-based exercise routines are time-efficient forms of exercise. When considering the structure of the exercise, if the total work is maintained consistently, the sequence of variable intervals does not affect the total exercise session EE, sRPE, or EES.	Instructors are recommended to administer mixed-intensity sessions to maintain constant energy demand, differentiating sessions to avoid boredom and promote adherence to training.
Ratajczak et al. (2020) (26)	Provide evidence on the impact of Spinning on the reduction of cardio-metabolic risk factors.	Cycle ergometer (Kettler DX1 Pro, Kettler, Ense, Germany).	Spinning is promising to reduce cardiometabolic risk factors, especially dyslipidemia. After 12 weeks of regular spinning, the metabolic function of the OW group was adapted in many ways to be more like that of the NW group.	The use of spinning as an effective modality in cardiac rehabilitation suggests that it can be as effective as standard training to improve exercise tolerance and cardiovascular health indicators.
Canário-Lemos et al. (2020) (27)	Determine if the use of HR and the rating of perceived exertion are effective means to control the intensity of Spinning classes and quantify their association with oxygen absorption.	Portable aerobic metabolic car (Cosmed K4, Rome, Italy); radiometer (Sanny AD 1010, American Medical do Brasil, Ltd. a, São Paulo, Brazil); HR monitor (Wireless Double Electrode, Polar®, Kempele, Finland).	Both the HR and the perceived effort classification are effective for controlling the intensity of Spinning classes in experienced subjects.	Instructors and coaches can use RPE in trained subjects as a cheaper and more effective way to control the intensity of spinning sessions.
Barbado et al. (2018) (28)	Examine the effects of sex, age, previous experience, the use of an HR monitor, the estimated sweating rate, and increases in room temperature and humidity in HR and RPE during Spinning sessions.	Suunto Team Pod (Vantaa, Finland) to register the average HR. Robertson OMNI 18 Scale.	The actual (%HRmax) and perceived (RPE) intensity of the exercise in a Spinning session is influenced by factors such as the estimated sweating rate or the increase in temperature or relative humidity produced in the cycling room.	HR monitoring and RPE are effective tools to guide the intensity of training in Spinning sessions. In addition, instructors must ensure optimal temperature and humidity conditions in the cycling room along with adequate hydration of the subjects.
Luszczyk et al. (2018) (29)	Evaluate the effects of spinning exercise compared to constant resistance exercise on fat oxidation and postexercise oxygen consumption.	Cycle ergometer (ViaSprint 150P; Ergoline, Bitz, Germany); cycle ergometer (Monark Ergomedic 839 E, Monark Exercise AB, Sweden.	These data indicate that spinning induces higher metabolic responses during the recovery period, and in the most effective way changes the pattern of use of the substrate toward lipids compared to the exercise of isocaloric resistance.	In the design of weight loss programs, spinning can be incorporated as an exercise modality that favors the oxidation of fats after exercise, being effective in improving body composition.
Rendos et al. (2015) (30)	Examine the effects of 3 body positions and 4 levels of RPE on cardiorespiratory response and normalized electromyographic activity of the vastus lateralis (NrmsEMGVL).	Spinning NXT (Star Trac, Irvine, CA. USA); portable ergo spirometry (Oxycon Mobile, Hoechberg, Germany); (Grass S88 Stimulator; Grass Medical Instruments, Quincy, MA, USA. USA) Biodex System 2 (Biodex Corp., Shirley, NY. USA).	Running and SC provide the greatest cardiorespiratory responses, and no maximum effort is needed for these responses. In addition, HR seems to be a bad indicator of oxygen consumption, especially in high RPE.	Body position and perceived effort have a significant impact on cardiorespiratory response and muscle activity during Spinning. This allows trainers to adjust the posture and intensity of the sessions to optimize the results of the training.
Soriano-Maldonado et al. (2014) (31)	Evaluate the effectiveness of an RPE learning protocol to improve the validity of the Borg 6-20 RPE scale for the intensity of the self-regulating exercise during Spinning.	IC BICYCLE (Keiser M3 Indoor Cycle, Fresno, California); Borg 6-20 RPE scale.	An RPE learning protocol could slightly improve the validity of the Borg 6-20 RPE scale for self-regulation of exercise intensity during spinning sessions in healthy adults.	An RPE protocol could be a useful tool for regulating the intensity of spinning sessions in healthy adults.
Ramos-Jiménez et al. (2014) (32)	Determine the physiological differences between the genders during physical exercise in Spinning with and without hydration.	Bicycle (Monark 828E, Vansbro Sweden); Digital infrared Ear 424 USA); heart rate (Polar Rs100, Finland); blood pressure (Aneroid Baumanometer and EM Rescue stethoscope, USA).	During hydration exercise (either with water or sports drink), the physiological response was similar for both sexes. Exercise without hydration produced physical stress, which could be prevented with any of the fluids (normal water was enough). Gender differences in the physiological response to the gyrus (body temperature, mean blood pressure, and HR) can be explained in part by the distinctive physical characteristics of everyone.	Hydration is crucial to prevent physical stress during prolonged exercise. Men tend to have more intense physiological responses than women in terms of blood pressure and body temperature, which helps instructors structure their training sessions.
Muyor (2013) (33)	1) Determine the intensity of a Spinning session; 2) know the correlation between the perceived rating of perceived exertion (Borg and OMNI) and the % of HR reserve (%HRR) with categories; and 3) assess the validity of the RPE scales (Borg and OMNI) concerning HR and %HRR.	Cycling was used (BH®, BHDuke®, Spain); Polar RS400 (Polar®Vantage NV, Polar Electro Oy, Finland); Borg and OMNI RPE scales.	Spinning causes a high-intensity effort that could be inappropriate for some participants. The Borg and OMNI scales showed low validity in quantifying the intensity performed in the spinning sessions. It indicates the need to control the intensity of the effort with other instruments to improve efficiency and reduce the risk of overload in this activity.	The Borg and OMNI scales showed little reliability to quantify the intensity of spinning. It is recommended to use other tools to monitor the perceived effort in these sessions.
Muyor & López-Miñarro (2012) (34)	Measure the response of the HR in the Spinning session and determine the validity of the Borg scale of the rating of perceived exertion as a measure of the intensity of the exercise in subjects who have had a 6-month experience in the Spinning activity.	Keiser Millenium (Fresno, California); (Polar Vantage NV, Polar Electro Oy, Finland); the BORG 6-20 RPE.	The reduced validity obtained for the general RPE and the high intensity achieved in adults during the Spinning session suggest a greater importance of controlling the intensity of this activity for adult subjects.	The reduced validity obtained for the general RPE and the high intensity achieved in adults during the Spinning session suggest a greater importance of controlling the intensity of this activity for adult subjects.
López-Miñarro & Muyor (2010) (35)	Measure the HR response of novice subjects performing a 45-minute spin cycle (R) in Spinning, and determine the validity related to the perceived effort classifications (RPE) criterion as a measure of exercise intensity in novice subjects.	Keiser Millenium (Fresno, California); (Polar Vantage NV, Polar Electro Oy, Finland); the BORG 6-20 RPE.	The intensity during the Spinning class in beginner adults ranged from moderate to difficult. These data suggest that indoor cycling should be considered a high-intensity exercise mode for novice subjects.	The reduced validity obtained for the general RPE indicates that more studies should be done before using this scale to regulate the intensity of exercise in novice adults during the spinning class.
Kang et al. (2005) (36)	Compare the metabolic and perceptual responses between the exercise performed on CON and with a VAR rotation protocol.		An exercise regimen in which the intensity varies does not exert any additional effect on metabolic and perceptual responses during exercise, if the average intensity remains the same. However, VAR resulted in higher O_2_ after exercise, and the increase in oxygen consumption by elevated plasma.	VAR exercise protocols can increase postexercise oxygen consumption, which is relevant to improving aerobic capacity and recovery in training programs.

PE, physical exercise; SVV, stroke volume variability; ACP, aerobic capacity plateau; HRV, heart rate variability; sRPE, perceived exercise session classification; EE, energy expenditure; RPE, rating of perceived exertion; HR, heart rate; VT, ventilatory threshold; HRmax, maximum heart rate; SC, standing climb; VAR, variable intensity.

## 3 Results and discussion

The outcome measures included in the current systematic review were those evaluated by at least 5 of the 17 included articles. These variables were i) physiological variables and spinning; ii) cardiac factors and spinning; iii) biomechanical considerations regarding the practice of spinning; and iv) perceived exertion and spinning.

### 3.1 What are the most commonly studied physiological variables in spinning among adults?

In the work of Battista et al. (12), observations of the intensity of spinning were made to document the intensity of the exercise. The results indicate that the simulated spinning classes produced an exercise intensity that varied significantly, with moderate average intensities of 74 ± 14% and 66 ± 15% of the VO_2max_ in classes 1 and 2, respectively. However, the momentary VO_2max_ exceeded the VO_2max_ during 10 of the 40 classes studied, with an average of 5.4 ± 4.5 min per session in these cases. Although the highest VO_2_ reached during classes (2382 ± 384 ml·min−1) was lower than the VO_2max_ recorded during the incremental test (2570 ± 341 ml·min−1), exercise during classes occasionally exceeded the observed maximum capacities. The classes were perceived as intense, with a RPE greater than 5, which suggests that, despite not reaching the maximum levels measured during the incremental test, the intensity of the spinning was considerable. As such, the data indicates that spinning should be considered a high-intensity exercise mode that has implications for both effectiveness and risk. These findings are supported by a study that evaluated differences in surface electromyography (sEMG) variables, HR, and perceived exertion in sedentary participants during a spinning session and compared them with those of trained subjects, demonstrating that the muscles of the trained participants are better adapted to physical exertion and experience less fatigue than those of the sedentary participants (14).

Similarly, Cortis et al. (25) reported that although a lower energy expenditure (EE) was observed during recovery in the descending protocol (DI), the intensity sequence of the intervals did not affect the total EE, physiological markers or perceptual responses during cycling. This relates to the study conducted by Bianco (16), which reports that HR at rest de-creased by 6.5% and 9%, respectively, after 24 and 36 sessions. After the tenth week, found a reduction of 11 beats.min−1 in the average training HR, an increase of 0.5 ml·kg−^1^·min−^1^ in the average oxygen uptake during training, and an increase of 8.6 watts in the average output power. In addition, an increase in cardiorespiratory fitness (37.1 ± 4.3 versus 40.2 ± 4.6 ml·kg−^1^·min−^1^) was observed after 36 sessions.

Additionally, Lai et al. (24) discussed physiological responses during incremental exercise in cycling. The temporal analysis revealed that the HR increased linearly in most participants, without exceeding the calculated maximum HRmax. The stroke volume (SV) reached its peak in the sixth stage and then decreased, directly influencing the in-crease in cardiac output in control group (CG) throughout exercise. The average values of three minutes per stage are presented, highlighting significant increases in the average HR (from 87.87 to 148.96 bpm), a similar pattern in the average SV, and an increase in the average CG (from 7086.86 to 10,480.96 ml/min). These findings suggest that, during incremental exercise, physiological responses such as HR, stroke volume, and cardiac output exhibit predictable patterns of adaptation and performance. This may be because the metabolic adaptations produced by two types of spinning protocols one of variable intensity and the other of constant intensity do not result in differences in mean VO_2max_ and HR during exercise in physically active adults (36), although it may have other metabolic effects. These findings are based on a study (29) that evaluated the effects of spinning compared to steady-state resistance exercise on fat oxidation and post-exercise oxygen consumption. The results revealed that, during the recovery period, compared with constant resistance exercise, spinning exercise resulted in a greater rate of lipid oxidation, as evidenced by a significant increase in the ratio of carbohydrate to fat (FAT/CHO). In addition, the VO_2max_ and respiratory ratio (RERmax) reached average values of 45 ± 2 ml·kg−^1^·min−^1^ and 0.85 ± 0.02, respectively, during the spinning exercise (29).

The results indicate that the actual HRmax and RPE of exercise in a spinning session are influenced by factors such as the estimated sweating rate or the increase in temperature or relative humidity produced in the cycling room (28). This is confirmed in the study conducted by Ramos-Jiménez et al. (32) where body temperature (*p* < 0.01), average blood pressure (*p* < 0.01), and HR (*p* < 0.01) were greater during exercise without hydration than during exercise with hydration, with no significant differences between the types of hydrations.

### 3.2 What is the role of spinning in cardiac rehabilitation programs?

A study in which a cardiac rehabilitation training program using spinning was conducted evaluating the effectiveness of this form of training over a 24-day period in patients who had suffered a myocardial infarction revealed a significant improvement in physical capacity (*p* < 0.001) and metabolic equivalents (*p* < 0.001) following the program (28). These results are supported by a study in which, after 24 days of a rehabilitation program, there was a significant improvement in physical capacity compared to the results obtained before the start of the program; in particular, there was a significant increase in test duration (9.21 ± 2.02 vs. 11.24 ± 1.26 min; *p* < 0.001 and 9.41 ± 0.39 vs. 10.91 ± 2.22 min; *p* < 0.001, respectively) (37). Although evidence regarding spinning suggests that this practice may be beneficial, the effectiveness of rehabilitation programs needs to be confirmed in future research. In this context, a study evaluating whether adding an eccentric cycling exercise program to a conventional cardiac rehabilitation program revealed that, although such practices are safe and well-tolerated by individuals, further studies are needed that account for the different conditions and characteristics of various populations (38).

### 3.3 What biomechanical considerations are relevant to spinning?

Research into the effects of pedaling cadence and rider position on initial power output and pedaling asymmetry during spinning reveals that, during standing intervals, power output was lower (132.4 ± 72.6 W vs. 197.5 ± 53.5 W; *p* < 0.05), and the bilateral leg asymmetry index was higher (52.2 ± 76.6% vs. 12.4 ± 9%; *p* < 0.05) than when participants pedaled in the seated position at a similar work intensity. In contrast, higher power outputs (238.1 ± 46.3 W vs. 153 ± 52.7 W; *p* < 0.05) and lower asymmetry indices (30.4 ± 39.2% vs. 12.6 ± 11%; *p* < 0.05) were recorded at intervals of 75 revolutions per minute (rpm) compared to 120 rpm, despite similar exercise intensities (39). The results indicate that, at similar training intensities, standing while cycling during a spinning class generates fewer watts and greater asymmetry than cycling while seated (39). This may be related to body position. In that context, a study examining three body positions and four levels of perceived exertion on cardiorespiratory response reveals that position plays a fundamental role in the perception of exertion, leading to increases in respiratory rate (30). This is confirmed by a study that sought to determine whether the saddle adjustment protocol plays a significant role in biomechanical measurements during pedaling, confirming that the protocol had a main effect on relative saddle height (*p* < 0.05) and knee length (*p* < 0.05) (40). The results indicate that the methods commonly used to ad-just saddle height are not appropriate for female indoor cyclists (only 20%–30% were adjusted correctly) and are moderately suitable for male cyclists (47%–60% were adjusted correctly). This may be due, in part, to sex differences in height and 3-D pelvic structure, as well as to the equations proposed in the literature, which were formulated only for male cyclists (41, 42).

### 3.4 What information do perceive exertion and pedalling provide?

Muyor (33) had two purposes: 1) to determine the intensity of a spinning session; 2) to determine the correlation between the RPE (Borg and OMNI) and the percentage of HR reserves (%HRR) with categories; and 3) to evaluate the validity of the RPE scales (Borg and OMNI) with respect to HR (HR) and %HRR. In this sense, the main phase was 152.2 ± 14.11 b·min−1, with a %HRR of 80.62 ± 7.10, which indicates a high intensity of exercise. The Borg and OMNI RPE scales averaged 14.94 ± 1.11 and 7.18 ± 0.79 points, respectively, during this phase. However, the correlation between average HR and %HRR with the Borg and OMNI scales was less than r < 0.4 (*p* < 0.05), suggesting a low validity of these scales to quantify the intensity of exercise in Spinning. The correlation coefficient between the Borg scale and OMNI scale was 0.82 (*p* < 0.001). Similarly, Muyor & López-Miñarro (34) measured the HR (HR) in the Spinning session to determine the validity of the Borg scale of the RPE as a measure of exercise intensity in subjects who had 6 months of experience in Spinning activity. The average HR in the cardiovascular phase was 144.11 ± 13.8 bmp. The mean values of the HR reserve percentage (% HRR) and general RPE in the cardiovascular phase were 77.8 ± 7.2% and 14.3 ± 1.7 points, respectively. The correlation value between the general RPE and %HRR was r = 0.18 (*p* > 0.05).

Canário-Lemos et al. (27) determined whether the use of cardiorespiratory fitness (CF) and the classification of perceived effort are effective means to control the intensity of spinning classes and quantify their association with oxygen absorption. During IC sessions, a significant effect of time on both VO_2max_ absolute and relative was observed (*p* < 0.001) for absolute oxygen consumption (VO_2abs_) and (*p* < 0.001) for VO_2max_ relative, indicating a considerable impact on oxygen consumption during sessions. In addition, a significant time‒session interaction was detected for VO_2max_ absolute (*p* = 0.014), and a session effect was detected for both VO_2max_ absolute and VO_2max_ relative (*p* < 0.001) for VO_2max_ absolute and (*p* = 0.001) for VO_2max_ relative). VO_2_ levels were significantly higher in the VO_2max_ controlled session than in the HR-controlled and effort-perception sessions, suggesting that VO_2_ intensity control may be more physically demanding. In all the sessions, tracks 4, 7, and 10 presented significantly higher VO_2abs_ and relative oxygen consumption (VO_2rel_) levels (*p* < 0.05), whereas track 1 presented lower levels, reflecting variations in exercise intensity. In addition, significant correlations were observed between the sessions, with correlations of 0.986 between the VO_2max_ relative of the average VO_2max_ session and the RPE session, 0.977 between the VO_2rel_ of the average VO_2max_ session and the HR session, and 0.992 between the VO_2max_ relative of the average HR session and the RPE session (*p* < 0.001) for all, suggesting that the exercise intensity control methods are highly related and can be interchangeable to some extent to measure the workload (31). These findings are important for designing effective training programs that show how controlling exercise intensity impacts physiological performance during IC sessions.

### 3.5 Limitations and future prospects

This systematic review has several limitations. First, although narrowing the sample to 17 studies improved the coherence of the evidence base, the included studies remain heterogeneous in participant characteristics (e.g., sedentary versus trained individuals, healthy adults versus clinical populations), study aims, and outcome domains (physiological, cardiac, biomechanical, and perceptual); this heterogeneity limits the extent to which findings can be pooled or generalized across contexts and should be considered when interpreting the conclusions of this review. Second, there is a limitation associated with study design, primarily because most of the research did not consider effects based on longitudinal studies or controlled trials. Third, there is a limitation associated with the small sample sizes and the variety of instruments used across studies. Therefore, it is necessary to consider these design-related limitations and the potential impact of unmeasured variables in order to avoid bias and improve the methodological quality of the scientific evidence.

The main recommendations are associated with possible research questions that are still unresolved and motivated by the low number of documents with high-quality evidence. For example, future lines of research must consider the development of longitudinal, experimental, and multivariate studies that allow the establishment of causal relationships and correlations to determine the effects produced by the practice of spinning associated with other variables of psychological, nutritional, biomechanical, etc., (43). A second approach can focus on the study of spinning in youth, seeking to understand the developments and improvements of the physical condition in the adolescent population and with it, favor a practice that can benefit cardiorespiratory fitness, metabolic adaptation, and the reduction of risk factors.

## 4 Conclusions

Within the scope of the 17 studies retained in this review, spinning emerged as a high-intensity exercise mode consistently associated with substantial physiological demand, and in several studies with improvements in cardiorespiratory fitness and a reduction in cardiometabolic risk factors. These benefits have been reported in specific contexts, such as cardiac rehabilitation programs and weight-management interventions in women with overweight, suggesting favorable metabolic adaptations; however, the marked heterogeneity in participant characteristics, study aims, and outcome domains across the included studies means these findings should not be generalized beyond the populations and contexts in which they were observed. On the other hand, the intensity of this activity could involve risks for untrained or sedentary people, or those with preexisting medical conditions, which calls for careful monitoring and adaptation of training to individual characteristics. Some reviewed studies reveal that common practices in equipment adjustment, such as saddle height, are not always adequate, which could affect the safety and effectiveness of exercise, especially in women. In addition, perceived exertion and HR have proven to be useful tools for controlling exercise intensity; however, the validity of the Borg scale may be limited. Overall, while the available evidence points to several potential benefits of spinning, the heterogeneity of the retained studies warrants caution in drawing broad conclusions about its effectiveness, and proper monitoring and consideration of individual capabilities remain crucial to maximizing training benefits while minimizing associated risks.

## Data Availability

The original contributions presented in the study are included in the article/Supplementary Material, further inquiries can be directed to the corresponding author.
